# Inducible and Reversible *Clock* Gene Expression in Brain Using the tTA System for the Study of Circadian Behavior

**DOI:** 10.1371/journal.pgen.0030033

**Published:** 2007-02-23

**Authors:** Hee-Kyung Hong, Jason L Chong, Weimin Song, Eun Joo Song, Amira A Jyawook, Andrew C Schook, Caroline H Ko, Joseph S Takahashi

**Affiliations:** 1 Howard Hughes Medical Institute, Northwestern University, Evanston, Illinois, United States of America; 2 Center for Functional Genomics, Northwestern University, Evanston, Illinois, United States of America; 3 Department of Neurobiology and Physiology, Northwestern University, Evanston, Illinois, United States of America; 4 Department of Psychology, University of Toronto, Toronto, Ontario, Canada; Stanford University School of Medicine, United States of America

## Abstract

The mechanism of circadian oscillations in mammals is cell autonomous and is generated by a set of genes that form a transcriptional autoregulatory feedback loop. While these “clock genes” are well conserved among animals, their specific functions remain to be fully understood and their roles in central versus peripheral circadian oscillators remain to be defined. We utilized the in vivo inducible tetracycline-controlled transactivator (tTA) system to regulate *Clock* gene expression conditionally in a tissue-specific and temporally controlled manner. Through the use of *Secretogranin II* to drive tTA expression, suprachiasmatic nucleus– and brain-directed expression of a tetO::*Clock^Δ19^* dominant-negative transgene lengthened the period of circadian locomotor rhythms in mice, whereas overexpression of a tetO::*Clock^wt^* wild-type transgene shortened the period. Low doses (10 μg/ml) of doxycycline (Dox) in the drinking water efficiently inactivated the tTA protein to silence the tetO transgenes and caused the circadian periodicity to return to a wild-type state. Importantly, low, but not high, doses of Dox were completely reversible and led to a rapid reactivation of the tetO transgenes. The rapid time course of tTA-regulated transgene expression demonstrates that the CLOCK protein is an excellent indicator for the kinetics of Dox-dependent induction/repression in the brain. Interestingly, the daily readout of circadian period in this system provides a real-time readout of the tTA transactivation state in vivo. In summary, the tTA system can manipulate circadian clock gene expression in a tissue-specific, conditional, and reversible manner in the central nervous system. The specific methods developed here should have general applicability for the study of brain and behavior in the mouse.

## Author Summary

Although significant progress has been made in unraveling the molecular mechanism of circadian clocks in mammals, previous work has focused on germline mutations and in vitro methods for analysis. To address the function of clock genes, it is necessary to develop tools to manipulate circadian genes in a conditional and tissue-specific manner in vivo. We report such an approach using the tetracycline transactivator system. Despite the development of the “tet” system in transgenic mice over 10 y ago by Bujard and colleagues, there are still relatively few examples of the successful use of the tet system in the central nervous system. Transgenic expression of the *Clock* gene in the suprachiasmatic nucleus and brain of mice regulated the period length of circadian locomotor rhythms. These effects could be inhibited by low doses of doxycycline in the drinking water. Importantly, low, but not high, doses of doxycycline were completely reversible and led to a rapid reactivation of the *Clock* transgenes. In summary, the tetracycline-controlled transactivator system can manipulate circadian clock gene expression in a tissue-specific, conditional, and reversible manner in the central nervous system. The specific methods developed here should have general applicability for the study of brain and behavior in the mouse.

## Introduction

Most organisms possess an endogenous circadian system that drives the daily timing of many physiological and behavioral processes. Genetic screens, spontaneous mutants, and gene-targeting approaches have been key in unraveling the essential set of genes underlying the circadian mechanism in mammals, *Drosophila,* and other model systems [1–4]. At the molecular and biochemical levels, a set of core clock genes govern positive and negative autoregulatory feedback loops of transcription and translation to form the core mechanism of the circadian clock in mammals [2,5]. The central oscillator is primarily driven by two bHLH-PAS transcription factors within the positive feedback loop, CLOCK and BMAL1, which heterodimerize and transactivate downstream clock and clock-controlled genes by binding to E-box elements that lie within their promoters [6–9]. The core constituents of the negative feedback loop are the *Cry* and *Per* genes, which are transcriptionally driven by CLOCK and BMAL1. PER and CRY proteins accumulate, associate with each other in the cytoplasm, translocate to the nucleus, and inhibit the CLOCK and BMAL1 activation of their own transcription [9]. As the negative elements turn over, CLOCK and BMAL1 renew their cycle of transcription of the *Per* and *Cry* genes.

In mammals, nearly all cells in the body contain circadian oscillators organized in a hierarchical fashion, with a master pacemaker located in the suprachiasmatic nucleus (SCN) of the anterior hypothalamus [5,10,11]. The SCN is entrained to the 24-h daily light–dark cycle via retinal light input and, in turn, synchronizes and coordinates the rhythms of peripheral tissue clock cells [2,5]. In mammals, luciferase reporters of circadian genes [10,11] in conjunction with single cell imaging have been valuable in revealing self-sustained circadian oscillators in virtually every cell in the body [12–15]. These studies have shown that most peripheral organs and tissues can express circadian rhythms in isolation; however, inputs from the dominant circadian pacemaker in the SCN are essential in coordinating circadian rhythms in an intact animal [10,11,16,17]. For example, SCN transplant experiments have shown that SCN-lesioned arrhythmic animals and genetically arrhythmic mice take on the rhythm of the donor SCN [17–19]. Similarly, transplanted mouse embryonic fibroblasts exhibit a circadian period and phase characteristic of the host rhythm and phase [20]. These findings have led to a widely accepted hierarchical model of the mammalian circadian system in which the SCN acts as pacemaker that drives and synchronizes peripheral circadian oscillators. Thus, understanding the physiological and functional relationships among central and peripheral clocks is essential; however, we still do not fully understand how the SCN governs peripheral oscillators to regulate circadian rhythms in physiology and behavior in multicellular organisms.

In nonmammalian systems, such as in *Drosophila,* analyses of the regulatory interactions of circadian genes led to the exploitation of novel tools to drive circadian genes, such as tissue-specific expression of transgenes (TGs) and reporters, which are valuable in elucidating the complexity of circadian system [21–23]. Conditional systems utilizing heat shock promoters have been developed in order to drive the temperature-dependent expression of circadian TGs [24–26]. Exogenous promoters in conjunction with the GAL4-UAS bipartite transgenic system have been valuable in expressing circadian TGs in subregions of the brain or distinct groups of circadian neurons, and even in ablation of discrete circadian neurons [27–30]. In mammals, however, other than ubiquitous inactivation of circadian genes by gene targeting techniques in embryonic stem cells or studies using the culture/explant-based system, the use of tissue-specific conditional regulation of circadian genes has not been reported [2,3]. Thus, to elucidate cellular and behavioral networks in the mammalian circadian system, more refined approaches are required, especially those affording temporal and spatial control of gene expression in vivo.

The ability to regulate TG expression in a conditional manner has made the tetracycline-controlled transactivator (tTA) system an attractive tool for use in mammalian systems. The tTA system was originally developed by Bujard and colleagues for the conditional expression of reporter genes in mammalian cells [31–34] and has been successfully applied in many experiments to study a variety of developmental processes and brain function in mammals [35–45]. The first component of this system contains a tissue-specific promoter that drives the expression of tTA, a fusion of the *Escherichia coli* tetracycline repressor sequence to the C-terminal transactivation domain of the herpes simplex virus VP16 gene that converts the repressor into a transcriptional activator. Expression of the target TG by tTA is achieved by introducing the TG of interest downstream of a minimal cytomegalovirus promoter sequence linked to multiple copies of the tet operator (tetO) sequence. Conditional and inducible regulation of the target TG is contingent on the ability of tTA to bind to the tetO sequences and activate transcription in a tetracycline-dependent manner. TG expression can be turned off with the administration of doxycycline (Dox), a tetracycline derivative, which prevents binding of tTA to the tetO sequence. Thus, unlike the site-specific recombinase Cre-*loxP* and Flp-*FRT* systems [46,47], which only allow conditional and/or tissue-specific inactivation of genes [48], the tTA system permits repeated cycles of conditional activation and inactivation of genes within the same animal. Hence, the tTA system provides *truly conditional* investigation of gene function. Despite the widespread use of the Tet system to manipulate various genes in a variety of tissues in mammals, only a few studies have used the system in the brain, and even fewer have used the system to study regulation of behavior [35,37–39,41–43,45,49]. A limitation of the tTA system in the study of brain function in vivo has been the slow induction of the TG (i.e., reversal) upon removal of standard dose of Dox [49].

Here we show that regulation of *Clock* ^Δ19^ or *Clock^wt^* TG expression occurs with rapid kinetics of induction and repression, causing an immediate reversion of the mutant to wild-type (WT) phenotype, and vice versa, in a tissue-specific manner. We demonstrate that the CLOCK protein is an excellent indicator for the kinetics of Dox-dependent induction/repression in the brain. Using activity rhythms as an output, the circadian period length provides a daily readout of the transactivation state of the Tet system in vivo. The development of the tTA system for conditional TG expression in the brain/SCN will open new avenues of research to answer fundamental questions of mammalian circadian biology.

## Results

### Generation of Transactivator and Target Transgenic Lines

**Figure 1:**
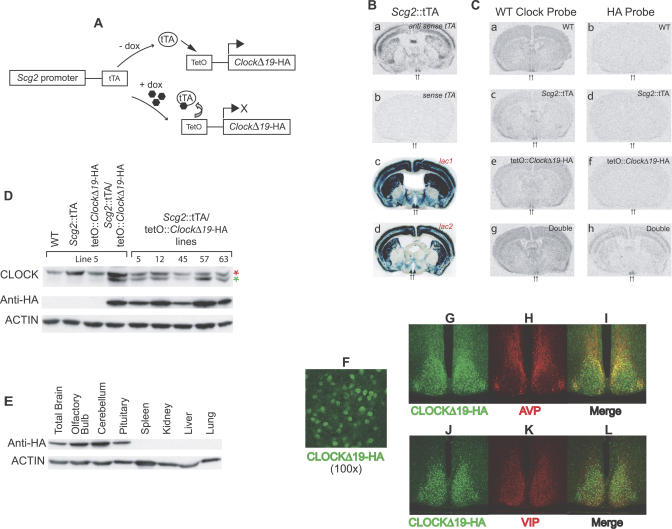
Regulation of the *Clock* ^Δ*19*^ TG Using the tTA System (A) Schematic diagram showing the Tet-Off system and constructs used for generating the tTA transactivator and target tetO transgenic lines. The *Scg2* promoter drives the expression of tTA, which binds to an array of cognate operator sequences in the absence of Dox but not in its presence, resulting in transcriptional activation or repression of the HA-tagged *Clock^Δ19^* TG, respectively. (B) Analysis of the *Scg2*::tTA line: (a) in situ hybridization of the tTA transcript, using an antisense tTA oligo probe, on coronal brain slices; (b) in situ hybridization of a control sense tTA oligo probe shows absence of specific hybridization and indicates that the antisense tTA oligo probe hybridization is specific; and (c and d) β-galactosidase staining of SCN/brain-specific induction of the *LacZ* TG in *Scg2*::tTA/tetO-*lacZ* mice, using two different tetO-*lacZ* reporter lines (lac1 and lac2). (C) In situ hybridization of endogenous WT *Clock* and HA-tagged *Clock^Δ19^* TG from *Scg2*::tTA × tetO::*Clock^Δ19^*-HA matings: representative coronal brain slices of (a and b) WT mice; (c and d) single transgenic *Scg2*::tTA mice; (e and f) single transgenic tetO::*Clock^Δ19^*-HA mice; and (g and h) double transgenic *Scg2*::tTA/tetO::*Clock^Δ19^*-HA mice. (D) Western blot analysis of transgenically induced CLOCK^Δ19^ and endogenous WT CLOCK in cerebellar lysates from all four possible genotypes. For the double transgenic mice, total brain lysates from five of the independent lines are shown with their line identity numbers indicated. Two independent mice from line 5 are also shown. The red asterisk indicates the WT protein, while the green asterisk denotes the HA-tagged CLOCK^Δ19^. (E) Western blot analysis of HA-tagged CLOCK^Δ19^ of various tissues from the double transgenic mice. (F–L) Immunocytochemical analysis of *Scg2*::tTA/tetO::*Clock* ^Δ*19*^ *-*HA mice. (F) Nuclear location of the CLOCK^Δ19^-HA. SCN/coronal sections were labeled with anti-HA antibody to detect transgenically induced CLOCK^Δ19^-HA (×100 magnification). (G–I) SCN/coronal sections were double labeled to detect transgenically induced CLOCK^Δ19^-HA (green, G) and endogenous vasopressin (AVP) (red, H). (I) Overlay of CLOCK^Δ19^-HA and AVP expression. (J–L) Double labeling of transgenically induced CLOCK^Δ19^-HA (green, J) and endogenous VIP (red, K). (L) Overlay of CLOCK^Δ19^-HA and VIP expression. Cells that express both the CLOCK^Δ19^ *-*HA and VP/VIP are shown in yellow in overlaid figures. (G–L) Captured with a ×20 objective.

The tTA (also known as Tet-Off) system requires two independent lines of transgenic mice (Figure 1A): one line expresses tTA under the control of a specific promoter, and a second line carries a tTA-responsive tetO promoter linked to the target gene of interest [31–34]. When two TGs are introduced into a single mouse through mating, the tetO-linked gene is activated but only in those cells that express tTA. Expression of the target TG can be suppressed by Dox.

To generate an SCN/brain-enriched transactivator line, we first searched for SCN-enriched transcripts that have expression restricted to the brain from the comprehensive mouse tissue expression databases [50,51]. This analysis revealed about 35 candidate genes that were either very highly expressed in the SCN or SCN/brain enriched. We then analyzed these candidate genes for SCN expression using in situ hybridization. From this screen, we found one gene, *Secretogranin II (Scg2),* that has very high constitutive expression in the SCN and has an expression pattern limited to brain, pituitary, and adrenal glands [52]. *Scg2* is a member of the neuroendocrine/neuronal secretory proteins, which are widely distributed in endocrine, neuroendocrine, and neuronal cells [53,54]. Although the exact role of *Scg2* is not well understood, research suggests that *Scg2* plays biologic roles in neurotransmission and paracrine regulation of central and peripheral actions of the nervous and neuroendocrine systems [54,55]. Because of its brain-enriched pattern of expression and, in particular, its strong expression in the SCN, we explored *Scg2* as a tissue-specific activator for the tTA system. We generated an SCN/brain-enriched transactivator line using a 9.8-kb promoter region of *Scg2.* We examined the pattern of expression using an oligo probe specific to the tTA transcript and showed distinct and strong tTA expression in the SCN but also throughout the brain (Figure 1Ba and 1Bb). To characterize tTA expression further, we crossed the *Scg2*::tTA line to mice carrying a tetO promoter-*lacZ* reporter construct [35]. We tested two independent tetO promoter-*lacZ* reporter lines, namely lines lac1 and lac2 (kindly provided by Mark Mayford, Scripps Research Institute, San Diego, California, United States). Both reporter lines showed similar SCN/brain-enriched expression patterns as demonstrated by β-galactosidase staining (Figure 1Bc and 1Bd). We used the *Scg2*::tTA line for all subsequent experiments described in this report.

Next, we generated the target line, which carries a tetO promoter fused to a dominant-negative *Clock* ^*Δ19*^ mutant allele [56,57]. In order to clearly assess the inducible expression of the TG, we also fused a hemagglutin (HA) epitope tag on the 3′ end of the cDNA, which does not interfere with CLOCK transcriptional activation [58]. We produced eight independent tetO::*Clock^Δ19^-*HA transgenic lines, and all lines were crossed to *Scg2*::tTA mice to evaluate whether we could control the expression of the TG in a temporal manner.

### Restricted Expression of the tetO-Linked *Clock^Δ19^* TG

We assessed whether the *Clock^Δ19^*-HA TG is expressed in *Scg2*::tTA/tetO::*Clock* ^*Δ19*^-HA double transgenic mice by in situ hybridization using oligo probes targeted to exon 19 of the *Clock* gene and to the HA tag (Figure 1C). The original ENU-induced *Clock^Δ19^* mutation is an A-to-T transversion in a splice donor site, causing skipping of exon 19 [57]; thus, the exon 19-specific probe detects only the endogenous WT *Clock* transcript. Conversely, the HA tag-specific probe detects only the *Clock^Δ19^*-HA TG transcript. All mice showed endogenous WT *Clock* transcript expression as expected. However, while only double transgenic *Scg2*::tTA/tetO::*Clock^Δ19^*-HA mice showed clear hybridization with the HA tag-specific probe, single transgenic (*Scg2*::tTA or tetO::*Clock^Δ19^*-HA) and WT mice did not show any expression of the HA tag. Thus, expression of the *Clock^Δ19^*-HA TG, which is evident in the SCN and throughout the brain, is induced by tTA in the double transgenic mice only. Similarly, Western blot analyses showed that only the double transgenic mice expressed the mutant TG protein (Figures 1D and S1A). Anti-CLOCK antibody detects both the mutant and WT proteins, which can be discriminated by their size difference. The lack of exon 19 in the mutant allele results in the deletion of 51 amino acids and subsequently produces a shorter protein for the *Clock^Δ19^*-HA TG compared to the WT allele. All eight independent tetO::*Clock^Δ19^*-HA transgenic lines expressed the mutant protein when crossed to *Scg2*::tTA mice (Figures 1D and S1A). In addition, the mean density ratio measurement of the WT and mutant proteins in Western blot analyses revealed that expression of the mutant protein is 0.51- to 1.05-fold relative to the WT protein in these lines. This was also demonstrated by Southern analysis (unpublished data), which showed that target lines carried approximately one or two copies of the TG. In order to test for leakiness of the tetO promoter, we inspected whether the tetO::*Clock^Δ19^-*HA single transgenic mice from each independent line expressed the TG using the anti-HA antibody against the brain lysates (Figure S1B). Of the eight independent target lines, only one (line 12) showed activation of the TG (likely due to a position effect on the TG), and therefore this line was excluded from further analysis. Thus, we obtained seven independent tetO target lines that showed tightly regulated inducible expression.

We next assessed tissue specificity and spatial expression of the *Clock^Δ19^*-HA TG in double transgenic mice. Western blot analyses indicated that expression of the TG is brain and SCN enriched (Figure 1E). Because *Scg2* participates in the neuroendocrine system [54,55], as expected, the TG was also expressed in the pituitary gland (Figure 1E); however, there was no expression in other peripheral tissues, such as the kidney, lung, liver, and spleen. Using fluorescence microscopy and an HA tag antibody, we explored the spatial expression of the TG within the SCN and found that *Clock^Δ19^*-HA TG expression is nuclear (Figure 1F). In WT mice, CLOCK has been shown to be nuclear and is expressed constitutively in the mouse SCN [59]. In contrast, rhythmically expressed negative factors of the circadian gene, such as PER1, PER2, and CRY1, accumulate in the nuclei at the end of circadian night [59,60]. To delineate further the spatial organization of the HA-containing cells within the SCN, we compared HA expression with the distribution of neuropeptide markers (Figure 1G–1L). Classically, the SCN is organized into two major subdivisions: a core that lies adjacent to the optic chiasm and is composed of neurons that predominantly produce vasoactive intestinal polypeptide (VIP) and a shell that surrounds the core and contain a large population of neurons that produce arginine vasopressin (AVP) [61–64]. Examination of the HA-containing cells showed that more than 90% of the cells in the SCN express the TG and that expression occurs throughout both the core and the shell. Serial sections through the rostral-caudal extent of the SCN showed that HA-expressing cells colocalized with AVP- and VIP-expressing cells (Figures 1F–1L, S2, and S3). Indeed, all AVP- and VIP-positive cell bodies in the SCN were also HA positive. Taken together, the detailed evaluation using Western blot and immunocytochemical analyses demonstrates that *Scg2-*driven *Clock^Δ19^*-HA TG expression is tissue specific and enriched in the brain and in the majority of cells in the SCN, including the core and the shell.

### Regulation of Conditionally Expressed *Clock^Δ19^* Allele by Doxycycline: Changes in Behavioral Circadian Rhythms

**Figure 2:**
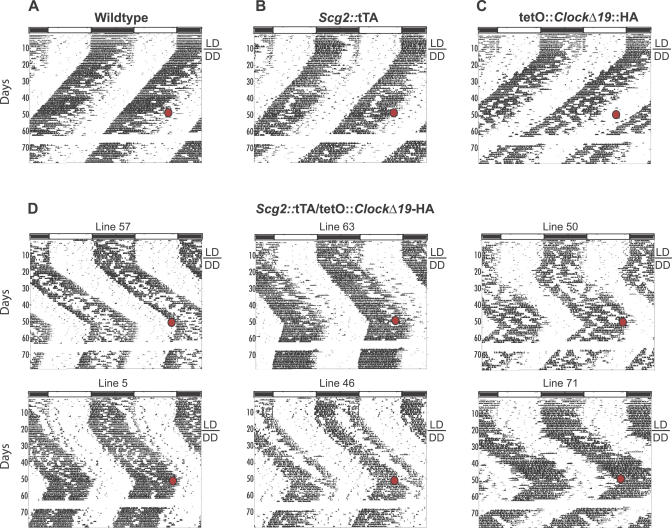
Locomotor Activity Records of Progeny from *Scg2*::tTA x tetO::*Clock* ^Δ*19*^-HA Matings: Representative Actograms Actograms are of (A) WT, (B) single transgenic *Scg2*::tTA, (C) single transgenic tetO::*Clock* ^Δ*19*^-HA, and (D) double transgenic *Scg2*::tTA/tetO::*Clock* ^Δ*19*^-HA mice. Activity records were double plotted so that 48 h is represented horizontally, with each day presented beneath and to the right of the preceding day. Wheel-running activity is indicated by black markings. The initial light cycle is depicted at the top of the record. All animals were maintained on a 12/12-h light–dark cycle for the first 12 to 14 d shown and then transferred to DD, as indicated by the bar next to each record. Numbers on the left indicate days of study. Six of the seven independent lines are shown. All double transgenic mice showed a lengthening of free-running period of about 1 h. Mice that carry a single TG only (*Scg2*::tTA or tetO::*Clock* ^Δ*19*^-HA) showed a free-running period similar to WT littermates. At the indicated red circle, all animals were given Dox (2 mg/ml) to “turn off” transgenically induced *Clock* ^Δ*19*^-HA TG. Only the double transgenic mice showed a shortening of free-running period, indicating suppression of the *Clock* ^Δ*19*^-HA TG expression. Period estimates during the Dox treatment showed wheel activity rhythm similar to WT mice. Administration of Dox in the single transgenic and WT mice showed no significant effects on the free-running period. Missing activity data (blank lines) on several concurrent days was due to computer malfunction.

The original *N*-ethyl-*N*-nitrosourea (ENU)-induced *Clock^Δ19^* mutant mice exhibit several behavioral alterations compared to WT mice, one of which is the lengthened free-running period of wheel-running activity. Heterozygous *Clock^Δ19^*/+ mice show a lengthening of the circadian period of approximately 1 h, while homozygous *Clock^Δ19^/Clock^Δ19^* mice exhibit periods about 4 h longer compared to WT mice and fail to express persistent circadian rhythms when maintained in constant darkness [65]. Because the *Clock^Δ19^* mutant allele behaves as an antimorph (one type of dominant-negative mutation), it competes with the WT allele in the generation of circadian rhythms [56,57,66]. In this study, because the *Scg2*::tTA/tetO::*Clock^Δ19^*-HA double transgenic mice express the *Clock^Δ19^*-HA TG as well as both copies of the endogenous WT CLOCK, we hypothesized that these mice would exhibit a free-running period of wheel activity rhythm similar to that of the heterozygous *Clock^Δ19^*/+ mice. To test this prediction, running-wheel behavior of all four genotypes from the *Scg2*::tTA x tetO::*Clock^Δ19^*-HA matings was recorded (Figure 2). All mice entrained to a 12/12-h light–dark cycle, initiating their nightly bouts of activity at the beginning of the dark phase with the majority of locomotor activity restricted to the night. Upon release into constant darkness (DD), however, only the double transgenic mice displayed a lengthened circadian period approximately 1 h greater than that of WT mice (23.9 to 24.8 h versus 23.4 to 23.7 h, respectively). Pairwise comparisons indicated that there were no significant differences in circadian period among the single transgenic lines (*Scg2*::tTA or tetO::*Clock^Δ19^*-HA) compared to WT littermates, while the period observed in double transgenic mice was significantly longer compared to the WT, *Scg2*::tTA, and tetO::*Clock^Δ19^*-HA mice (mean DD comparison among all genotypes, F_3,78_ = 118.77, *p* < 0.00005; each pairwise comparison [double transgenic versus others], *p* < 0.0005). Double transgenic mice from all seven independent tetO lines showed lengthening of the circadian period similar to the circadian period exhibited in the heterozygous *Clock* mutant mice. Therefore, conditional expression of the dominant-negative mutant allele TG in the SCN and brain is sufficient to drive altered running wheel activity behavior. As described above, these independent lines bear one or two copies of the TG, and each line displays slightly different expression levels (0.51- to 1.05-fold; Figure 1D). It is noteworthy that those double transgenic mice, which carry one or two copies of the *Clock^Δ19^* TG and two endogenous WT copies of *Clock*, display a free-running period similar to that of the *Clock* heterozygote mice *(Clock^Δ19^/+),* which carry one copy of each allele (mutant and WT *Clock*). (There was a trend toward longer circadian period in lines that expressed higher levels; however, the differences were subtle.) It is not surprising, therefore, that we do not observe a circadian rhythm phenotype similar to that of *Clock* homozygous-like behavior (i.e., 4-h lengthening and arrhythmicity) in double transgenic mice, given the relative magnitude of TG expression in relation to the expression of WT CLOCK.

**Figure 3:**
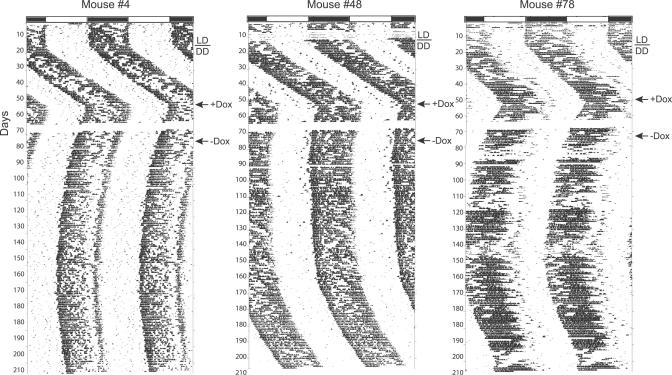
Persistent Effects of 2 mg/ml Dox Treatment on Circadian Activity Rhythms of *Scg2*::tTA/tetO::*Clock* ^Δ*19*^-HA Double Transgenic Mice Although the effect of Dox treatment was relatively rapid (+Dox, also see Figure 2), the treatment was not immediately reversible upon returning to water (−Dox) from 2 mg/ml Dox. It took at least 3 mo before the double transgenic mice returned to their original longer circadian period.

**Figure 4:**
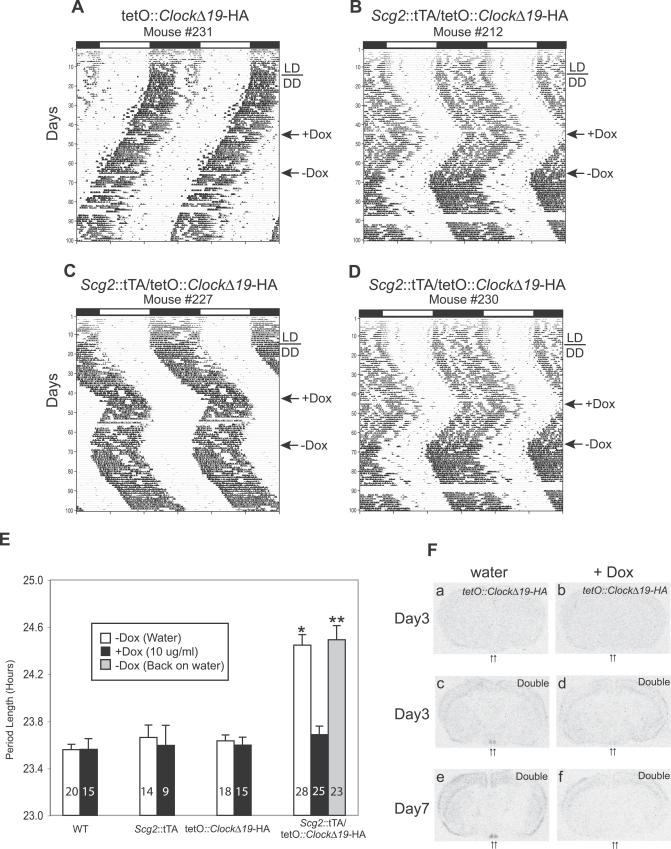
Rapid Inhibition and Reversal of tTA Mediated tetO::*Clock* ^Δ*19*^-HA Transcription in *Scg2*::tTA/tetO::*Clock* ^Δ*19*^-HA Double Transgenic Mice to a Lower Concentration of Dox Representative actograms of (A) a single transgenic tetO::*Clock* ^Δ*19*^-HA mice and (B–D) double transgenic *Scg2*::tTA/tetO::*Clock* ^Δ*19*^-HA mice on 10 μg/ml Dox treatment. Administration of 10 μg/ml Dox was just as effective as 2 mg/ml Dox on the free-running wheel period; all double transgenic mice showed a shortening of circadian period. Withdrawal of Dox (−Dox, second arrow) led to reversal back to the previous long circadian period by next day. (E) Comparison of free-running period estimates for all four genotypes. Data are presented as mean with corresponding 95% confidence interval. Numbers on the bottom of the bars indicate the number of animals in each group that were wheel tested for their locomotor activity rhythm. Overall, there were significant differences in mean circadian period among genotypes (F_3,78_ = 118.77, *p* < 0.00005). Pairwise comparisons indicated that there were no significant differences on circadian period in single transgenic lines (*Scg2*::tTA or tetO::*Clock* ^Δ*19*^-HA) compared to the WT littermates; however, a significantly longer free-running period was observed in double transgenic mice compared to WT, *Scg2*::tTA, and tetO::*Clock* ^Δ*19*^-HA (*p < 0.0005 for each comparison), indicating that the transgenically induced *Clock* ^Δ*19*^-HA TG expression causes this period difference. With administration of 10 μg/ml Dox, no difference was observed among the genotypes on circadian period (F_3,62_ = 1.93, *p* = 0.1339), thus the transgenically induced *Clock* ^Δ*19*^-HA TG expression was “turned off.” Rapid reversal was achieved when the double transgenic mice were returned to water treatment (−Dox); their free-running period reverted back to the previous circadian period, lengthening about 1 h (**paired *t*-test, *t*_*22*_ = −17.2177, *p* < 0.00005). (F) In situ hybridization of the *Clock* ^Δ*19*^-HA transcript on coronal brain slices from the single tetO::*Clock* ^Δ*19*^-HA and double transgenic mice (*Scg2*::tTA/tetO::*Clock* ^Δ*19*^-HA) on water (−Dox, left side) and 10 μg/ml Dox (+Dox, right side) treatments, respectively. TG transcript was detected using an antisense HA oligo probe. The transgenically induced *Clock* ^Δ*19*^-HA transcript is “turned off” by day 3 on 10 μg/ml Dox treatment.

One advantage of tetracycline controlled gene expression is that a gene of interest can be repeatedly turned on and off noninvasively. To examine Dox-dependent transactivation, we treated all mice initially with 2 mg/ml Dox in their drinking water (Figure 2). Upon treatment, the *Scg2*::tTA/tetO::*Clock^Δ19^*-HA double transgenic mice showed a shortening of circadian period in about a day, indicating a rapid suppression of *Clock^Δ19^*-HA TG expression. Period estimates during the Dox treatment showed a mean circadian period of 23.7 h (SD = 0.196, *n* = 16) in double transgenic mice, similar to their WT littermates (23.6 h, SD = 0.108, *n* = 22). Next, we examined whether the effect was reversible by removing Dox from the drinking water after 3 wk of treatment. The circadian period of double transgenic mice, however, did not immediately return to the previous long circadian period; rather, it took longer than 3 mo for reversal (Figure 3). One possibility for this long and gradual reversal of the circadian period may be due to period “after effects”, a form of behavioral plasticity in which the free-running period of circadian behavior undergoes experience-dependent changes, such as, in this case, exposure to Dox [67,68]. However, Dox is a potent effector substance for the tetracycline-controlled gene expression system and it is also lipophilic in nature; thus, a likely possibility is that the dosage used may have been too high to achieve rapid clearance and subsequent rapid reversal. Earlier studies have shown that 2 mg/ml Dox produces a rapid switch-off of TG expression (within 1 d) in many tissues and organs [69–71]; however, after 1 mo of treatment, clearance of the antibiotic can take as long as 6 wk [71]. Consequently, we tested lower Dox concentrations in the range of 10 ng/ml to 100 μg/ml and found that 10 μg/ml Dox was optimal. Similar to 2 mg/ml Dox treatment, the *Clock^Δ19^*-HA TG was rapidly turned off at 10 μg/ml (Figure 4); double transgenic mice showed a shortening of their circadian period of about 1 h, while single transgenic or WT mice showed no effect of Dox treatment. No difference in circadian period was observed among the genotypes with 10 μg/ml Dox treatment (F_3,62_ = 1.93, *p* = 0.1339). More important, rapid reversal (switching-on) was achieved when the double transgenic mice were returned to water; their period lengthened by approximately 1 h within one or two cycles (paired *t*-test, *t*_*22*_ = −17.2177, *p* < 0.00005). In addition, in situ hybridization revealed that *Clock^Δ19^*-HA transcript in the SCN and throughout the brain is sensitive to the presence of 10 μg/ml Dox. By day 3 of treatment, the transcript is not detectable. Therefore, a dose of 10 μg/ml Dox in the drinking water can easily cross the blood-brain barrier and is sufficient to rapidly switch expression of the TG on and off in the brain. In addition, we found that 100 μg/ml tetracycline in the drinking water also worked effectively (Figure S4). The magnitude and ease by which we can alter circadian wheel running behavior using a low-dose provide flexibility of repeated activation and repression. Importantly, our data argue that the circadian period is regulated through the dynamic and daily expression of *Clock* and *Clock*-controlled genes rather than through a static process established during embryonic development.

### Regulation of Conditionally Expressed *Clock^Δ19^* Allele by Doxycycline: Changes in Behavioral Response to Discrete Pulses of Light

**Figure 5:**
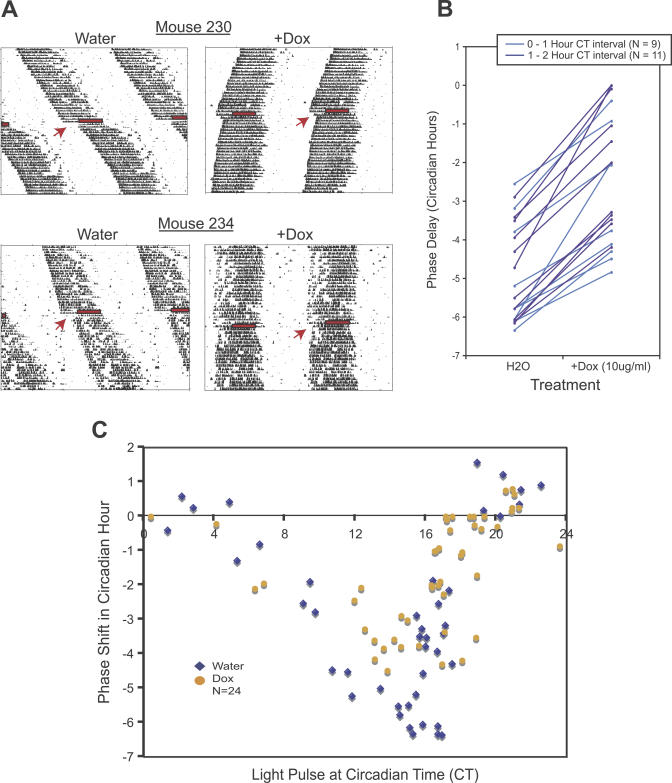
Light-Induced Phase Shifting Responses in *Scg2*::tTA/tetO::*Clock* ^Δ*19*^-HA Double Transgenic Mice (A) Two examples of locomotor activity records of double transgenic mice, where they received a 6-h light pulse, during no treatment (water, expression of *Clock* ^Δ*19*^-HA TG) and during Dox treatment (+Dox, repression of *Clock* ^Δ*19*^-HA TG). All light pulse experiments were carried out after the animals were released into DD. For mouse 230, a 6-h light pulse was given at CT 16 on water treatment and at CT 16.4 on Dox treatment. For mouse 234, a 6-h light pulse was given at the same CT of 16.7 on water and on Dox treatments. The red arrow indicates the time at which the mice were light pulsed. (B) Comparison of light-induced phase shifts in 20 individual double transgenic mice following a 6-h light pulse either during water or 10 μg/ml Dox treatment. Data shown represent animals that received light pulses between CT 12 to CT 18. The symbol on the left side indicates phase delay of each mouse after a light pulse on water treatment (*Clock* ^Δ*19*^-HA TG expression on). The symbol on the right side indicates phase delay of the same mouse on Dox treatment (*Clock* ^Δ*19*^-HA TG expression off). Animals were light pulsed at a given CT during water treatment and then the animals were later treated with Dox to turn off the TG. Animals were light pulsed again at the closest CT as possible to the original light pulse, within 1-h window (blue lines) or within 1- to 2-h window (purple lines). All double transgenic mice show a larger phase shift after a light pulse with water treatment, when the TG is on, compared to with Dox treatment, when the TG is turned-off (for 0- to 1-h CT interval, paired *t*-test, *t*_8_ = 6.6026, *p* = 0.0002; for 1- to 2-h CT interval, paired *t*-test, *t*_10_ = 11.5582, *p* < 0.00005). (C) PRC to discrete pulses of light in *Scg2*::tTA/tetO::*Clock* ^Δ*19*^-HA double transgenic mice. The *x*-axis indicates the CT at the beginning of the light pulse. The *y*-axis indicates the phase shift (in hours) produced by the light pulse. When the *Clock* ^Δ*19*^-HA TG is on, the double transgenic mice showed a characteristic increased amplitude PRC, similar to the behavior seen in heterozygous *Clock^Δ19^* mutant mice after discrete pulses of light; however, when the TG is off, the PRC in double transgenic mice is similar to behavior observed in WT mice.

Light remains one of the most well-understood circadian entraining signals and perturbation analyses with light has been exploited to demonstrate functional properties of the circadian clock [72]. The phase-response curve (PRC) can be considered a fundamental pacemaker property [67]. Exposure to light early in the night phase shifts the clock so that subsequent cycles begin at a later time; however, exposure to light late in the night advances the circadian clock. These time-dependent responses to light are important for synchronization to environmental light conditions. Another altered behavioral phenotype in *Clock^Δ19^*/+ mice is high-amplitude phase-resetting responses to a 6-h light pulse (type 0 resetting) compared to WT mice, which exhibit low-amplitude (type 1) phase resetting [73]. In the C57BL/6J inbred strain of mice, a 6-h light pulse near the “break-point” (the transition from phase delays to phase advances at approximately circadian time [CT] 17) produces large phase shifts of about 4 to 6 h; however, *Clock* heterozygotes display phase shifts of longer than 6 h [73]. In order to assess whether the conditional induction of *Clock^Δ19^*-HA TG, restricted to brain/SCN, recapitulates behavioral properties of *Clock^Δ19^*/+ heterozygous mice, we chose to characterize the phase shifting response. Within the same animal, when *Clock^Δ19^*-HA TG is expressed (or when the mice are on water), their phase shift is significantly larger than when the TG is turned off (when they are on Dox treatment) (Figure 5A). The amplitude of phase shift in *Scg2*::tTA/tetO::*Clock^Δ19^*-HA double transgenic mice on Dox treatment is comparable to the phase shift/delay described in WT mice after a 6-h light pulse [73]. During subjective night (between CT 12 and CT 17), double transgenic mice showed a significantly larger phase delay on water compared to Dox treatment (Figure 5B). The difference between the two treatments (water versus Dox) is more clearly demonstrated when the data are presented as a PRC (Figure 5C). Therefore, brain/SCN-specific activation of the *Clock^Δ19^*-HA TG affects both the phase resetting, as well as, the period lengthening effects on locomotor activity rhythms, similar to that seen with the original *Clock^Δ19^*/+ heterozygous mutation in the whole animal.

### Conditional Regulation of the *Clock^wt^* Allele

**Figure 6:**
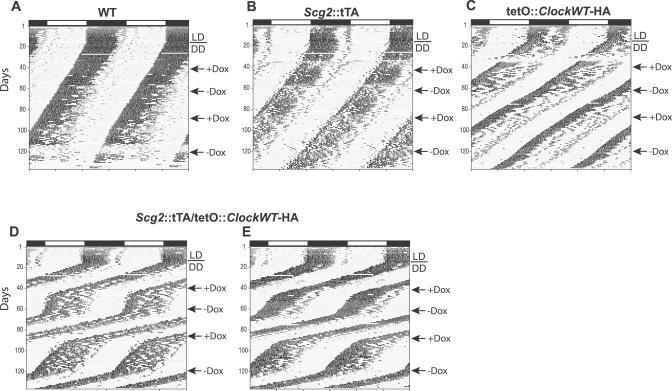
Locomotor Activity Records of Progeny from *Scg2*::tTA × tetO::*Clock^wt^* -HA Matings: Representative Actograms Actograms of (A) WT, (B) single transgenic *Scg2*-tTA, (C) single transgenic tetO::*Clock^wt^*-HA, and (D and E) double transgenic *Scg2*::tTA/tetO::*Clock^wt^*-HA mice. All double transgenic mice, expressing the *Clock^wt^* -HA TG, showed a shortening of circadian period of approximately 1 h, beyond the normal WT values. Mice that carry only a single TG (*Scg2*::tTA or tetO::*Clock* ^Δ*19*^-HA) showed circadian period range similar to their WT littermates. At the first arrow, all animals were given Dox (10 μg/ml) to turn off transgenically induced CLOCK^wt^-HA. At the second arrow, Dox was withdrawn to switch back on the *Clock^wt^*-HA TG. A similar effect on circadian period was observed when Dox was administered and withdrawn for the second time in the same animal.

In our previous report on the rescue of the *Clock* mutation using bacterial artificial chromosome (BAC) TGs, we showed that overexpression of WT *Clock* allele (*Clock^wt^*) shortened free-running period length beyond the WT range [66]. Thus, we explored whether a conditional expression of *Clock^wt^*, restricted to the SCN and brain, could also generate shortened free-running activity rhythms as observed in transgenic mice that overexpress CLOCK^wt^ ubiquitously. We have crossed one of the tetO::*Clock^wt^*-HA target lines to the *Scg2*::tTA activator line, and progeny from the mating were examined for their locomotor activity rhythm. We find that the *Scg2*::tTA/tetO::*Clock^wt^*-HA double transgenic mice exhibit a shortened circadian period by about 1 h compared to the WT circadian period (Figure 6). In addition, similar to transcriptional activation of the *Clock^Δ19^*-HA TG, we demonstrate temporal control of *Clock^wt^*-HA TG expression with rapid switch-off and a rapid reversal. Therefore, conditional expression of the dominant-negative mutant allele, as well as overexpression of the WT allele, restricted to the SCN and brain, is sufficient to alter the period of activity rhythms. Given that the peripheral circadian oscillators in these mice are WT, it will be interesting in future experiments to investigate whether the period of peripheral oscillators in these mice can be regulated as a consequence of period changes induced by the SCN/brain as previously suggested in experiments using transplanted mouse embryonic fibroblasts [20].

## Discussion

Significant progress has been made in unraveling the molecular mechanism underlying the mammalian circadian system. The core molecular circuitry of opposing interlocking transcriptional feedback loops has been defined as the fundamental basis of the circadian clock [2,74]; however, with subsequent discovery of additional molecular components of the circuitry [75–78], the complexity and network intricacy of the clock system are becoming apparent [2,11,50,79]. Ultimately, we want to understand how these cell-autonomous circadian oscillators interact in multicellular organisms to regulate physiology and behavior [80]. Thus, to elucidate the mechanisms governing the hierarchical nature of mammalian circadian timing, it is necessary to develop genetic tools to manipulate circadian genes in a conditional and tissue-specific manner in vivo.

We adapted an in vivo transgenic method, the tTA system, to regulate *Clock* gene expression in a conditional and reversible manner. This is a significant technical milestone that has not been previously demonstrated in the mammalian circadian system. In this report, we generated an SCN- and brain-enriched tTA-expressing transgenic line, which can transactivate any transcript of choice when crossed to tetO-responsive transgenic lines. We also produced target lines that can express a *Clock^Δ19^* mutant allele or *Clock^wt^* allele in a tissue-specific manner when crossed to a transactivator transgenic line. The *Clock^Δ19^* allele is a dominant-negative mutation (antimorph) [56] and is an ideal allele to validate the tTA system for circadian experiments for several reasons. First, the *Clock* gene, with its heterodimeric partner *Bmal1/Mop3,* is one of the primary transcriptional activators of the circadian transcriptional autoregulatory feedback loop. Second, the *Clock^Δ19^* mutation in mice perturbs the circadian system and causes a significantly altered running-wheel rhythm that is clearly distinguishable from WT [56,57,65,66]. The antimorphic nature of this mutation, which has a phenotypic effect that is antagonistic with the normal allele, allows manifestation of the mutant phenotype in the presence of the WT allele. Third, we have previously demonstrated not only that transgenic expression of WT *Clock* can completely rescue circadian period and amplitude in *Clock* mutant mice but also that overexpression of the *Clock^wt^* TG ubiquitously results in period shortening beyond the normal WT values [66]. Fourth, *Clock* gene expression in the SCN is constitutive, which is easier to mimic with tTA system. Finally, all circadian clock mutants that have been analyzed in mammals thus far have been germline mutations (either ENU-induced or gene targeted), and therefore the developmental consequences of these mutations have not been addressed. For all of these reasons, we set out to test conditional expression of *Clock* on circadian behavior.

Interestingly, the circadian periodicity of *Scg2*::tTA/tetO::*Clock^Δ19^*-HA double transgenic mice provides a real-time readout of the transactivation state of this system. This allowed us to follow the kinetics of Dox regulation on a day-to-day basis and revealed that high doses of Dox (2 mg/ml) required many months of time to washout and reverse. This then gave us the opportunity to find optimal Dox dose treatments for both inactivation and reversal of tet-dependent transactivation in the brain of mice. SCN-directed expression of the dominant-negative *Clock^Δ19^* TG lengthens the circadian free-running period, whereas expression of the *Clock^wt^* TG shortens the circadian rhythm. Furthermore, we showed that temporal and spatial control of TG expression can revert the phenotype from a mutant to a WT state within individuals, and vice versa, with a low concentration of Dox treatment, so that experiments can be performed in a longitudinal fashion. This is akin to transplant experiments [17–19] with the added ability to reverse the procedure. Moreover, unlike transplant experiments, which only allow for the receiving of intact humoral signals, our system also retains intact synaptic connections of the SCN. Finally, the expression of the TG affects not only the free-running activity rhythm but also other expected circadian behavioral responses, such as phase shifts to discrete light pulses. Thus, the transgenic mice described in this report are a valuable tool and will facilitate investigation of the functional relationship between central and peripheral clocks.

The validation of the tTA system for conditional TG expression in a variety of cell types and tissues has made it the tool of choice for mammalian system research. Arguably, some of the most significant contributions were made by several groups utilizing the Tet-system in vivo to study the effects of conditional TG activation and repression on various neurobiological process [35–39,43,45,81,82]. However, our study differs uniquely from previous Tet system applications in several ways. First, this is the first report on the mammalian circadian system where an SCN/brain-driver is used to conditionally regulate clock genes in vivo. Only a handful of studies reported have used the Tet system in the brain and, thus, the availability of brain-specific drivers is very limited. Furthermore, no drivers have previously been shown to function in the SCN. Second, our study demonstrates that circadian locomotor activity records give us a unique opportunity to have a daily readout of the transactivation state in a noninvasive manner. We suspect that this is likely due to a combination of the shorter half-life of the target protein, CLOCK, and our optimization of the Dox dosage used. Thus, we show that CLOCK is an excellent indicator for the kinetics of Dox-dependent induction/suppression in the brain. Third, we show that the standard dose often used in Tet regulation studies (e.g., 2 mg/ml) is excessively high. This leads to very slow kinetics of washout and slow (weeks to months) reactivation of the TG after standard doses of Dox treatment [49]. To date, only one study has examined a time course of TG expression in the brain tissues using luciferase activity as an indicator of the TG expression and has suggested administering a 100-fold lower dose (50 μg/ml) [36]. In our study, we demonstrate that even a 200-fold lower dose of 10 μg/ml in drinking water is sufficient to cross the blood-brain barrier and is equally effective in turning off the TG, and subsequently regulate behavioral state. With a lower dose, our results reveal that the washout is rapid and, thus, multiple induction and suppression cycles of the TG can be achieved within subjects with minimal time loss and cost. Furthermore, the effectiveness of the low Dox dosage is not driver specific. We have found that another SCN- and brain-enriched driver to be inducible and reversible using the same low dose administration (unpublished data). Finally, this study provides an important set of transgenic mouse resources for the circadian research community.

Exploitation of these transgenic lines along with existing genetic allelic series of circadian genes may yield fundamental insights into the mechanism by which circadian pacemaker systems transmit information to control physiology and behavior. In addition, by using peripheral tissue-specific drivers, manipulations using the tTA system can yield a wealth of knowledge on physiological processes tied to the circadian machinery such as cell division, heme biosynthesis, tumor suppression, metabolism, and bone remodeling [83–87]. For example, we recently reported a dissection of tissue-specific functions of the mammalian clock protein BMAL1 using the SCN- and brain-enriched driver line, which we describe here, and a muscle-specific driver line. We showed that distinct tissue-specific phenotype in *Bmal1-*null mice can be rescued using the tTA system [88]. Moreover, tetracycline-dependent genetic tools can also assist in elucidating unexpected subtle phenotypes found in knockout mice of some essential clock genes, such as *Rev-erbα* and *Clock* [89,90], and address our current criteria for definition of primary clock components [3]. Besides being potentially useful for such studies, the tTA system may be a great resource for the discovery and in vivo validation of novel candidate genes that may be involved in the central SCN oscillator and the output pathway. The successful manipulation of conditional TG expression in the SCN and brain in these studies will lay the groundwork for the development and adaptation of other tools such as the Cre/Lox system for tissue-specific knockout and conditional inactivation of circadian genes. Furthermore, by developing additional SCN subregional-specific drivers, we can begin to decipher the function of the cellular heterogeneity of the mammalian SCN and to understand how these pacemaking neurons are organized to mediate synchrony within the SCN and the whole animal. The flexibility of the tTA system provides a means to dissect the cellular and behavioral networks in the mammalian circadian system.

## Materials and Methods

### TG constructs.

For the generation of tTA-expressing mice (transactivator line), a 1,153-bp fragment upstream of the translation start site of the mouse *Secretogranin II* gene was amplified by PCR primers 5′-AGTGATTCCTCTTACTAATCCATCTGTGAGAT-3′ (forward) and 5′-GTCTTAAAGATTTCCTGAAAACATAGA-3′ (reverse) using a mouse BAC clone RP23-470F8 as a template (Roswell Park Cancer Institute Mouse BAC Library; Invitrogen, http://www.invitrogen.com). The PCR product was first cloned into the EcoRV restriction site in pBluescript II SK(−) (pBlue) (Stratagene, http://www.stratagene.com). This plasmid was then cut with EcoRI and was used to ligate a 9-kb EcoRI promoter fragment isolated from the BAC clone RP23-470F8, resulting in an intact 9,851-bp promoter fragment upstream of the translation start site. This plasmid was named pBlue-*Scg2*promoter. A 1,453-bp EcoRI-XmnI fragment coding tTA was released from the PMY20 plasmid (kindly provided by Mark Mayford [35]) and ligated into the SalI restriction site of the pBlue-*Scg2*promoter plasmid, after T4 DNA polymerase treatment of both fragments. This plasmid was named *Scg2*::tTA and was linearized with NotI restriction enzyme prior to microinjection. The linearized fragment was isolated using the QIA Quick Gel Extraction Kit (Qiagen, http://www.qiagen.com) and dialyzed for 4 h in 10 mM Tris-HCl (pH 7.5)/0.1 mM EDTA (pH 8.5) buffer before pronuclear injection.

For the generation of tetO target lines, we first generated the 3′-HA–tagged WT *Clock* and mutant *Clock^Δ19^* cDNAs from the plasmids pBlue-ClockWT and pBlue-ClockΔ19, respectively, as described previously [57]. Each full-length cDNA also contained 388 bp of 5′ untranslated region of *Clock,* which was generated from exons 1b, 2, and 3. The following primers were used against the pBlue-ClockWT or pBlue-ClockΔ19 plasmid to generate cDNAs by PCR using Pfu DNA polymerase (Stratagene): 5′-ATAAGAATGCGGCCGCGGGGAGGAGCGCGGCGGTAGCGGTG-3′ (forward) and 5′-CCCAAGCTTCTAAAGAGCGTAATCTGGAACATCGTATGGGTACTGTGGCTGGACCTTGGAAGGGTCA-3′ (reverse). Amplified product was digested with NotI and HindIII, gel purified, and ligated into the NotI-HindIII cloning site of the pTRE2 plasmid (Clontech, http://www.clontech.com). These tetO::*Clock^wt^*-HA and tetO::*Clock^Δ19^*-HA pTRE2 plasmids were linearized using DrdI/XmnI restriction enzymes to exclude most of the vector backbone sequence prior to pronuclear injection. All plasmids were sequence verified using the ABI PRISM Dye Terminator Cycle Sequencing Ready Reaction Kit and analyzed on an ABI377 automated sequencer (Applied Biosystems, http://www.appliedbiosystems.com).

### Transgenic animals.

Transgenic mouse lines were generated by pronuclear injection using standard techniques [91] and essentially as described in Antoch et al. [66]. Briefly, the linearized DNA fragment was injected into fertilized mouse oocytes isolated from crosses of WT CD1 matings at a concentration of 1 ng/μl. Transgenic mice were identified by PCR analysis of genomic DNA prepared from tail biopsy samples. PCR amplification of the transactivator TG (*Scg2*::tTA) was carried out using primers (forward primer 5′-AGACAAGCTTGATGCAAATGAG-3′; reverse primer 5′-CAAGTGTATGGCCAGATCTCAA-3′) that generate a 482-bp fragment. Two *Scg2*::tTA founder lines were obtained and characterized, and one line was chosen for the experiments presented here. For the tetO::*Clock^wt^*-HA and tetO::*Clock^Δ19^*-HA TGs, genotyping was performed using 5′-ATATGCAAGGCCAGGTTGTC-3′ (forward primer) and 5′-TCTGTGGCATACTGGATGGA-3′ (reverse primer), which generates a 258-bp fragment. We produced eight tetO::*Clock^Δ19^*-HA lines and 13 tetO::*Clock^wt^*-HA target lines. Each founder animal was maintained as a congenic line by backcrossing to the C57BL/6J inbred strain for at least four generations. All animals were raised in a 12/12-h light–dark cycle from birth. All animal studies were conducted in accordance with the regulation of the Committee on Animal Care and Use at Northwestern University.

### Dox treatment.

Doxycycline hydrochloride (Sigma-Aldrich, http://www.sigmaaldrich.com) was supplied in the drinking water at a concentration of 2 mg/ml or 10 μg/ml. The Dox-containing water was renewed every 2 to 3 d. Mice were supplied with regular food and water with or without Dox ad libitum.

### Circadian behavioral analysis.

Wheel-running activity of singly housed animals was recorded and analyzed essentially as described [65]. Mice were entrained to a 12/12-h light–dark cycle for a minimum of 7 d before they were released into constant darkness. Activity data were collected as number of events per minute and recorded continuously by a PC system (Chronobiology Kit; Stanford Software Systems, http://www.query.com); data were analyzed using ClockLab software (Actimetrics, Wilmette, Illinois, United States). The free-running period was calculated (days 1 to 20 in DD) by using a χ^2^ periodogram [92]. For the light pulse experiments, a 6-h light pulse (approximately 100 lux) was provided at a given CT, where CT 0 denotes the beginning of the subjective day and CT 12 denotes the beginning of the subjective night. The magnitude of phase shift was determined by measuring the phase difference, based on the activity onset as a phase reference point, between regression lines fit immediately before the light pulse and at least seven consecutive activity-onset times after the light pulse (excluding the four cycles immediately after the pulse) as described previously [73]. The magnitude of phase shift was corrected for circadian period as estimated before the light pulse for each individual. All other statistical analyses were performed using the Stata Statistical/Data Analysis software (version Stata/SE 9.0; StataCorp, http://www.statacorp.com).

### Western blotting.

Tissues were homogenized in a buffer containing 150 mM NaCl, 50 mM Tris (pH 7.4), 1% Triton X-100, 0.1% SDS, and protease inhibitor cocktail (Complete; Roche Applied Science, http://www.roche.com). Homogenates were cleared by centrifugation at 10,000*g* for 10 min at 4 °C, and supernatants were collected and protein concentration was estimated using Bio-Rad DC Protein Assay (Bio-Rad, http://www.bio-rad.com) according to the manufacturer's instructions. Total protein (40 μg) was mixed with sample buffer according to the protocol of [93] and resolved on an 8% SDS–polyacrylamide gel by electrophoresis. Thereafter, proteins were electrotransferred onto a Poly Screen PVDF transfer membrane (Perkin Elmer Life Sciences, http://www.perkinelmer.com). The membranes were blocked with PBST (PBS + 0.1% Tween-20) containing 5% powder milk for 1 h and then incubated with the 3F10 mouse monoclonal anti-HA–peroxidase antibody (1:500; Roche Applied Science) or anti–actin-peroxidase antibody (1:1,000; Santa Cruz Biotechnology, http://scbt.com) according to the manufacturer's protocol. Anti-CLOCK guinea pig antibody (1:1,000) was followed by anti–guinea pig IgG secondary antisera horseradish peroxidase (1:1,000; Jackson ImmunoResearch Laboratories, http://www.jacksonimmuno.com). CLOCK guinea pig antibody was generously provided by Choogon Lee (University of Florida, Tallahassee, Florida, United States). Proteins were visualized with a chemiluminescence detection system (ECL Western blotting detection analysis system; Amersham Pharmacia, http://www.amersham.com) and with subsequent exposure to autoradiographic film.

### In situ hybridization.

In situ hybridization procedures were performed as described [94]. Briefly, animals were sacrificed by cervical dislocation; the brains were removed immediately, frozen on dry ice, and stored at −80 °C. Sectioning, fixation, hybridization, and washing were performed as described. Sections were hybridized using an antisense-HA oligo probe (5′- AAGAGCGTAATCTGGAACATCGTATGGGTACTGTGGCTGG-3′), a WT *Clock* oligo probe (5′-GCTCTAGCTGGTCTTTTAGATGCTGCATGGCTCCTAACTGAGCTG-3′), or an *Scg2* oligo probe (5′-TTCAGCAGCTCCAGGGCGGAGTTGATCACCTTGGACTTGTCCAGGCGGGACAT-3′). Primers were 5′-end-labeled with [γ-^33^P]ATP by using recombinant terminal deoxynucleotidyl transferase (Invitrogen).

### Immunocytochemistry.

Animals were anesthetized with ketamine/xylazine/saline cocktail (10 mg/ml ketamine, 2 mg/ml xylazine; Phoenix Scientific, http://www.psiqv.com) at 0.01 ml/g body weight and then perfused intracardially with 50 ml of 0.9% saline solution followed by 50 ml of 4% paraformaldehyde (Sigma Aldrich) in 0.1 M phosphate buffer (pH 7.2). The brains were removed and postfixed for 24 to 48 h at 4 °C in 4% paraformaldehyde in 0.1 M phosphate buffer. For immunocytochemistry, 50-μm coronal sections were collected through the entire SCN using a VibroSlice microtome (World Precision Instruments, http://www.wpiinc.com) and processed free floating. Sections were incubated with mouse anti-HA.11 biotin-labeled monoclonal antibody (BIOT-101L-100, 1:200; Covance Research Products, http://www.crpinc.com) followed by anti-biotin, mouse IgG1, monoclonal 2F5 conjugated with Alexa Fluor 488 secondary antibody (1:1,000; Invitrogen). A primary antibody to either VIP (1:5,000; ImmunoStar Inc, http://www.immunostar.com) or AVP (1:10,000 ImmunoStar Inc) was followed by Alexa Fluor 568 goat anti-rabbit IgG (H+L) secondary antibody (1:1,000; Invitrogen). The Alexa Fluor 488 signal was assigned a green pseudo-color, while the Alexa Fluor 568 signal was assigned a red pseudo-color. Sections were viewed with a Leica DMRXE7 confocal microscope with Ar (488 nm) and green HeNe (543 nm) lasers in the Biological Imaging Facility at Northwestern University (Evanston, Illinois, United States). Images were captured by sequential excitation with each laser to avoid crosstalk between the two wavelengths using the Leica Confocal Software (version 2.61 build 1537).

### β-Galactosidase staining.

Mice carrying a tetO promoter-*lacZ* reporter construct (lines lac1 and lac2) were generously provided by Mark Mayford. All procedures were performed as described by Low-Zeddies and Takahashi [80]. Briefly, mice were anesthetized with ketamine/xylazine/saline cocktail and then transcardially perfused with chilled 0.1% heparin PBS. Brains were removed and postfixed for 30 min in 4% paraformaldehyde PBS on ice and then stored overnight in 20% sucrose PBS at 4 °C. Brains were frozen on dry ice, embedded in Shandon Lipshaw M1 embedding matrix (Pittsburgh, Pennsylvania, United States), and sectioned coronally at 50-μm thickness. Free-floating sections were collected in a wash buffer (PBS with 2 mM MgCl_2_, 0.02% NP-40 [Sigma] [pH 7.3]) and then incubated for 24 h at 37 °C in an X-gal staining solution (1 mg/ml 5-bromo-4-chloro-3-indolyl-β-d-galactoside; Gold Biochemical, Long Beach, New York, United States) dissolved in dimethyl sulfoxide buffer containing 5 mM K_3_Fe(CN)_6_ and 5 mM K_4_Fe(CN)_6_). Finally, sections were rinsed three times in the wash buffer and then mounted in aqueous mounting medium (3:1 glycerol/PBS) on gelatin-coated glass slides. Stained sections were viewed and photographed under bright-field illumination.

## Supporting Information

Figure S1 Western Blot Analysis of *Scg2*::tTA/tetO::*Clock^Δ19^-*HA Double Trasngenic Mice (A) Western blot analysis of CLOCK in cerebellar lysates from *Scg2*::tTA/tetO::*Clock^Δ19^*-HA double transgenic mice of seven independent lines. The red asterisk indicates the WT protein, while the green asterisk denotes the HA-tagged CLOCK^Δ19^.
(B) Western blot analysis of CLOCK in cerebellar lysates from single transgenic tetO::*Clock^Δ19^*-HA mice of seven independent lines. Only one line (line 12) shows leakiness of the tetO promoter. The eighth independent line (line 71) also expressed the TG with a tight regulation (unpublished data).
(1.0 MB PDF)

Figure S2 Immunocytochemical Analysis of *Scg2*::tTA/tetO::*Clock^Δ19^-*HA Double Transgenic Mice Serial coronal sections from the rostral-caudal extent of the SCN were double labeled to detect transgenically induced CLOCK^Δ19^-HA (green) and endogenous AVP (red). Overlay of CLOCK^Δ19^-HA and AVP expression is shown in the right column. Figures were captured at ×20 magnification.
(1.8 MB PDF)

Figure S3 Immunocytochemical Analysis of *Scg2*::tTA/tetO::*Clock^Δ19^-*HA Double Trasngeneic Mice Serial coronal sections from the rostral-caudal extent of the SCN were double labeled to detect transgenically induced CLOCK^Δ19^-HA (green) and endogenous VIP (red). Overlay of CLOCK^Δ19^-HA and VIP expression is shown in the right column. Figures were captured at ×20 magnification.
(2.6 MB PDF)

Figure S4 Tetracycline Works as Well as Doxycycline for Reversal of tTA-Mediated tetO::*Clock^Δ19^*-HA Transcription in *Scg2*::tTA/tetO::*Clock^Δ19^*-HA Double Transgenic Mice Administration of 100 μg/ml tetracycline in the drinking water was just as effective as 10 μg/ml Dox in perturbing the free wheel-running period behavior; all double transgenic mice (*Scg2*::tTA/tetO::*Clock^Δ19^*-HA) show shortening of circadian period. A dosage of 100 μg/ml tetracycline was rapidly reversible after the withdrawal.
(1.9 MB PDF)

### Accession numbers

Accession numbers for genes used in this paper are mouse BAC clone RP23-470F8 (GenBank [http://www.ncbi.nlm.nih.gov/Genbank] AC084213), *Clock* (GenBank AF000998 [mRNA], AF146793 [genomic], Entrez Gene ID 12753, UniProt O08785), *Bmal1 (Arntl)* (Entrez Gene ID 11865, UniProt Q9WTL8), *Scg2* (Entrez Gene ID 20254), *Avp* (Entrez Gene ID 11998, UniProt P35455), and *Vip* (Entrez Gene ID 22353, UniProt P32648).

## Abbreviations

AVP: arginine vasopressin
BAC: bacterial artificial chromosome
CT: circadian time
DD: constant darkness
Dox: doxycycline
ENU: N-ethyl-N-nitrosourea
HA: hemagglutin
PRC: phase-response curve
SCN: suprachiasmatic nucleus
TG: transgene
tetO: tet operator
tTA: tetracycline-controlled transactivator
VIP: vasoactive intestinal polypeptide
WT: wild-type
