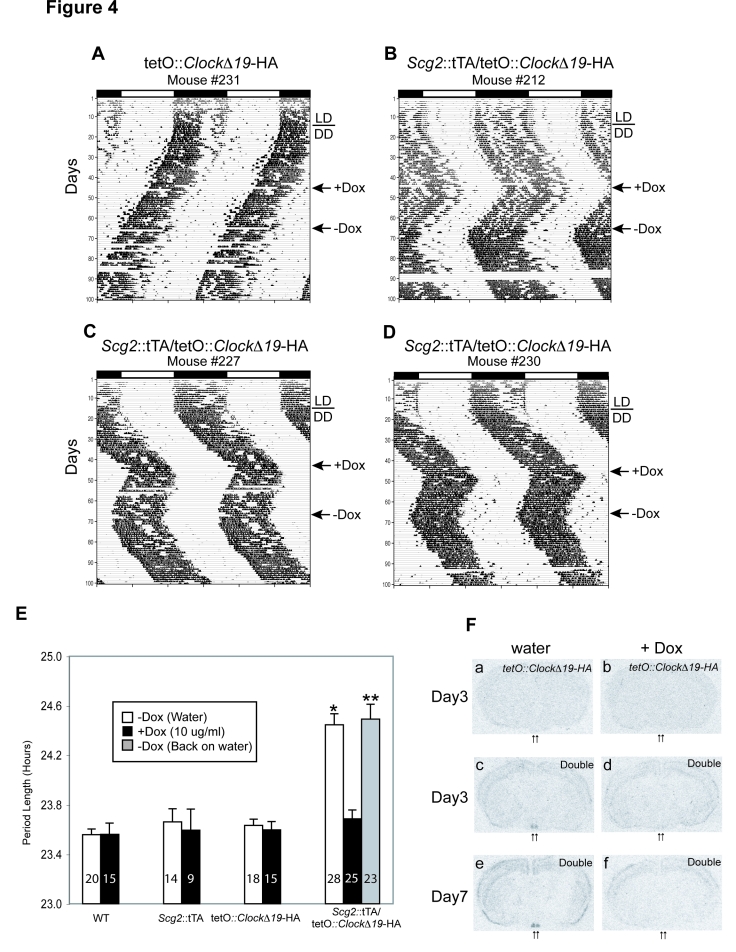# Correction: Inducible and Reversible *Clock* Gene Expression in Brain Using the tTA System for the Study of Circadian Behavior

**DOI:** 10.1371/annotation/cf732434-c0e7-40d0-8491-784193689056

**Published:** 2011-06-01

**Authors:** Hee-Kyung Hong, Jason L Chong, Weimin Song, Eun Joo Song, Amira A Jyawook, Andrew C Schook, Caroline H Ko, Joseph S Takahashi

Figure 4b is incorrectly duplicated in Figure 4d. Please view the correct Figure 4 here: 

**Figure:**