# Supplementary figures and images for: Inducible and Reversible Clock Gene Expression in Brain Using the tTA System for the Study of Circadian Behavior
Source: PLoS Genet. 2007 Feb 23;3(2):e33. doi: 10.1371/journal.pgen.0030033 (PMC1802832; doi:10.1371/journal.pgen.0030033)

## A

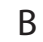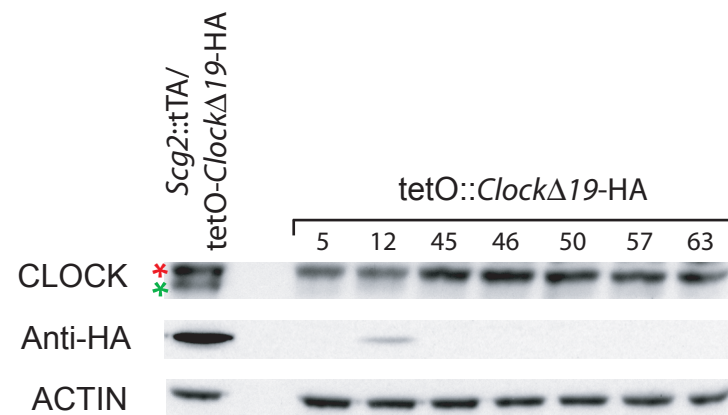

Supplement: Figure S1 — (A) Western blot analysis of CLOCK in cerebellar lysates from Scg2::tTA/tetO::ClockΔ19-HA double transgenic mice of seven independent lines. The red asterisk indicates the WT protein, while the green asterisk denotes the HA-tagged CLOCKΔ19. (B) Western blot analysis of CLOCK in cerebellar lysates from single transgenic tetO::ClockΔ19-HA mice of seven independent lines. Only one line (line 12) shows leakiness of the tetO promoter. The eighth independent line (line 71) also expressed the TG with a tight regulation (unpublished data). (1.0 MB PDF) [file pgen.0030033.sg001.pdf]

Supplemental figure 2

Rostral



Caudal

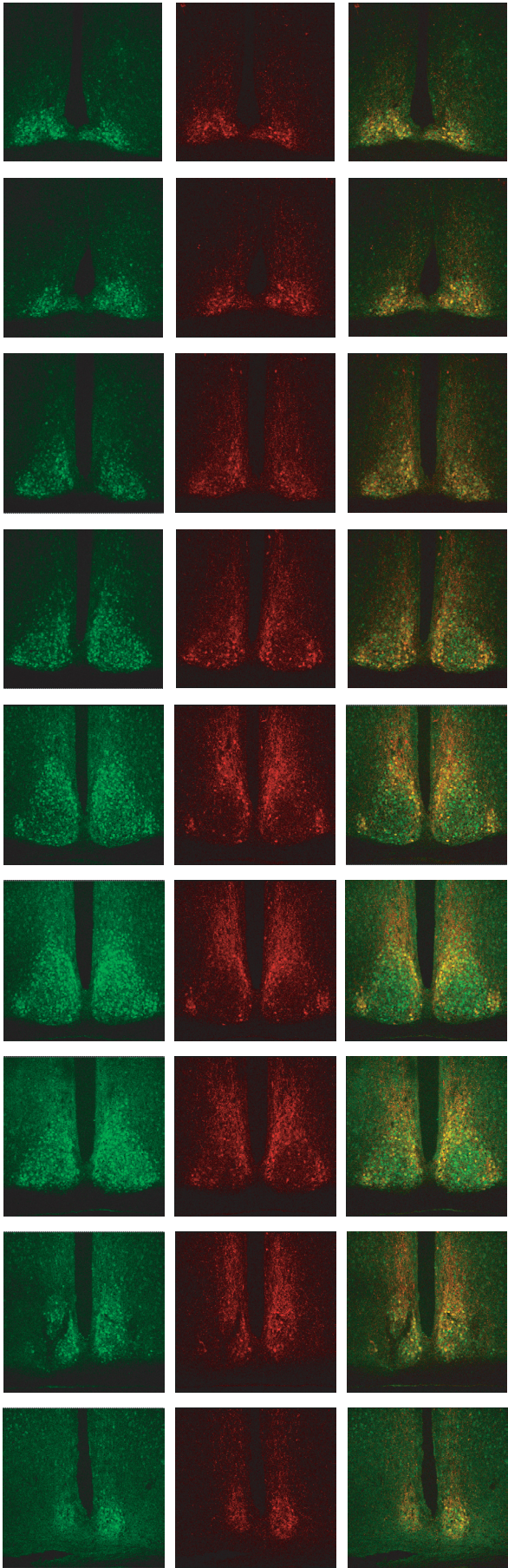

HA/AVP/Overlay  
ZT4  
20X

Supplement: Figure S2 — Serial coronal sections from the rostral-caudal extent of the SCN were double labeled to detect transgenically induced CLOCKΔ19-HA (green) and endogenous AVP (red). Overlay of CLOCKΔ19-HA and AVP expression is shown in the right column. Figures were captured at ×20 magnification. (1.8 MB PDF) [file pgen.0030033.sg002.pdf]

Supplemental Figure 3

Rostral

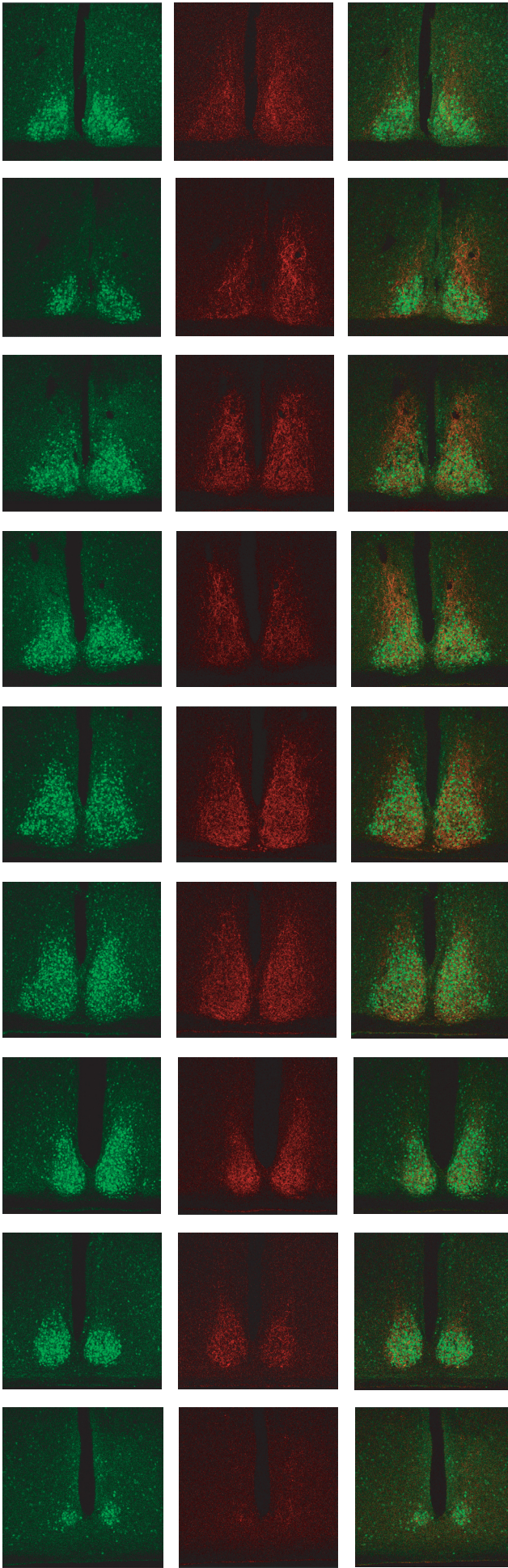

HA/VIP/Overlay  
ZT4  
20X

Caudal

Supplement: Figure S3 — Serial coronal sections from the rostral-caudal extent of the SCN were double labeled to detect transgenically induced CLOCKΔ19-HA (green) and endogenous VIP (red). Overlay of CLOCKΔ19-HA and VIP expression is shown in the right column. Figures were captured at ×20 magnification. (2.6 MB PDF) [file pgen.0030033.sg003.pdf]

Supplemental Figure 4

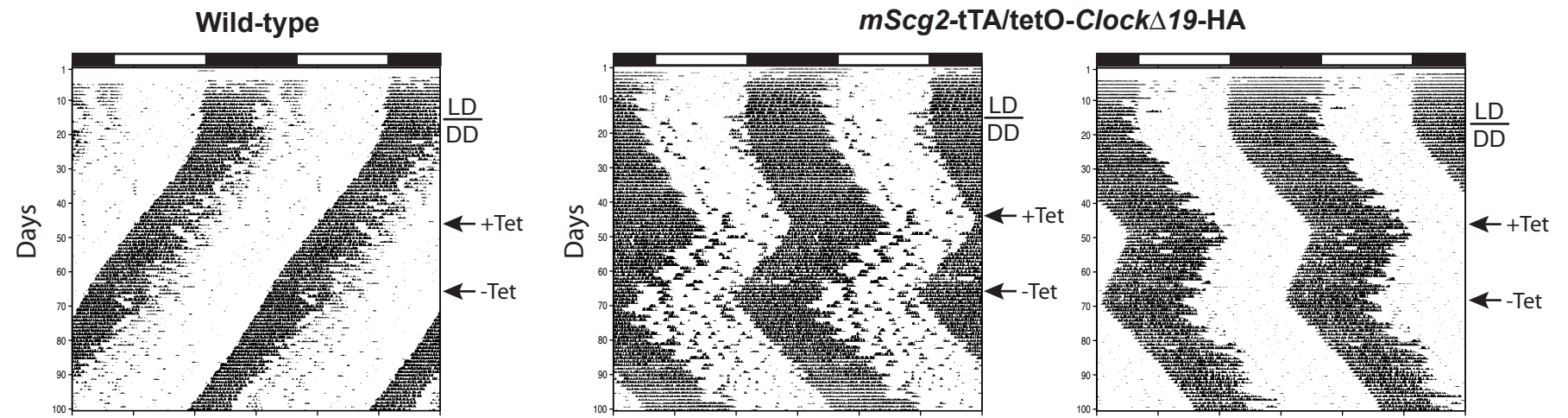

Supplement: Figure S4 — Administration of 100 μg/ml tetracycline in the drinking water was just as effective as 10 μg/ml Dox in perturbing the free wheel-running period behavior; all double transgenic mice (Scg2::tTA/tetO::ClockΔ19-HA) show shortening of circadian period. A dosage of 100 μg/ml tetracycline was rapidly reversible after the withdrawal. (1.9 MB PDF) [file pgen.0030033.sg004.pdf]
